# Angiocentric glioma in refractory epilepsy: when to suspect?

**DOI:** 10.1055/s-0045-1809659

**Published:** 2025-06-25

**Authors:** Angelo Dante de Carvalho Corrêa, Sérgio Ferreira Alves Júnior, Luis Alcides Quevedo Canete, Nina Ventura

**Affiliations:** 1 Instituto Estadual do Cérebro Paulo Niemeyer, Rio de Janeiro RJ, Brazil.; 2 Universidade Federal do Rio de Janeiro, Rio de Janeiro RJ, Brazil.

**Figure 1:**
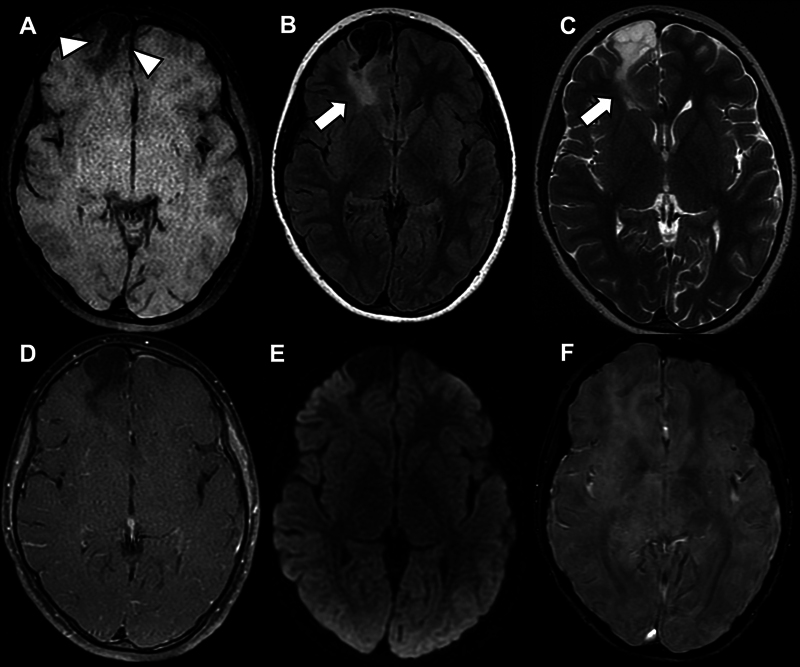
Non-contrast T1-weighted imaging (T1-WI) ( **A** ) showing an expansive right frontal lesion with faint areas of hyperintense signal in its periphery (white arrowheads), a feature commonly observed in angiocentric glioma (AG). Fluid-attenuated inversion recovery (FLAIR) ( **B** ) and T2-weighted imaging (T2-WI) ( **C** ) showing a band of hyperintense signal extending from the lesion to the right lateral ventricle (Stalk-like sign; white arrows), which may represent tumor growth along the vessels. These two findings, within the clinical context, favored the diagnosis of AG. Moreover, the lesion did not present contrast enhancement on postcontrast T1-WI ( **D** ), neither foci of restricted diffusion ( **E** ) nor foci of bleeding or calcification in the susceptibility-weighted imaging (SWI) ( **F** ), findings that are usually absent in AG.

A 9-year-old boy presented with partial seizures that had progressively worsened throughout the previous 4 months; the patient was refractory to oral medication. Magnetic resonance imaging (MRI) scans ( Figure 1 ) were performed, which demonstrated an expansive right frontal lesion, suggesting a tumor from the group of long-term epilepsy associated tumors (LEATs), with some findings suggestive of angiocentric glioma (AG), 1 2 confirmed after surgical resection.

A rare World Health Organization (WHO) grade 1. pediatric-type. diffuse low-grade glioma, 3 AG is an epileptogenic lesion often observed in young patients. 4 The imaging findings ( Figure 1 ) may be characteristic and lead to radiological suspicion before surgery, 1 2 which can be essential for correct patient management.
